# Acid Dentin Lysate Failed to Modulate Bone Formation in Rat Calvaria Defects

**DOI:** 10.3390/biology10030196

**Published:** 2021-03-05

**Authors:** Jila Nasirzade, Karol Alí Apaza Alccayhuaman, Zahra Kargarpour, Ulrike Kuchler, Franz Josef Strauss, Layla Panahipour, Carina Kampleitner, Patrick Heimel, Frank Schwarz, Reinhard Gruber

**Affiliations:** 1 Department of Oral Biology, Medical University of Vienna, 1090 Vienna, Austria; jila.nasirzaderajiri@meduniwien.ac.at (J.N.); caroline7_k@hotmail.com (K.A.A.A.); zahra.kargarpooresfahani@meduniwien.ac.at (Z.K.); ulrike.kuchler@meduniwien.ac.at (U.K.); franzstraussa@gmail.com (F.J.S.); layla.panahipour@meduniwien.ac.at (L.P.); 2 Department of Oral Surgery, Medical University of Vienna, 1090 Vienna, Austria; 3 Clinic of Reconstructive Dentistry, University of Zurich, 8032 Zurich, Switzerland; 4 Department of Conservative Dentistry, School of Dentistry, University of Chile, Santiago 1058, Chile; 5 Karl Donath Laboratory for Hard Tissue and Biomaterial Research, Medical University of Vienna, 1090 Vienna, Austria; carina.kampleitner@meduniwien.ac.at (C.K.); patrick.heimel@trauma.lbg.ac.at (P.H.); 6 Ludwig Boltzmann Institute for Experimental and Clinical Traumatology, 1200 Vienna, Austria; 7 Austrian Cluster for Tissue Regeneration, 1200 Vienna, Austria; 8 Department of Oral Surgery and Implantology, Johann Wolfgang Goethe-University, Carolinum, 60596 Frankfurt am Main, Germany; f.schwarz@med.uni-frankfurt.de; 9 Department of Periodontology, School of Dental Medicine, University of Bern, 3012 Bern, Switzerland

**Keywords:** autogenous tooth roots, bone regeneration, rat calvaria defect, collagen membranes, guided bone regeneration, bone augmentation

## Abstract

**Simple Summary:**

Tooth roots are increasingly applied for bone reconstruction before implant placement. Growth factors stored in the dentin are assumed to enhance bone regeneration, however, the evidence is low. To this aim, collagen membranes were coated with dentin lysates obtained from extracted porcine teeth or remain untreated. The collagen membranes were tested for their capacity to stimulate bone formation in rat calvarial bone defects. After four weeks of healing, micro-computed tomography and histological analyses revealed that dentin lysates coating had no significant impact on the rather strong bone regeneration reaching a nearly complete defect closure even in untreated defects. It can thus be concluded that dentin lysates do not hinder bone regeneration. Conclusions concerning a possible stimulation of bone regeneration by dentin lysates should not be drawn.

**Abstract:**

Autogenous tooth roots are increasingly applied as a grafting material in alveolar bone augmentation. Since tooth roots undergo creeping substitution similar to bone grafts, it can be hypothesized that osteoclasts release the growth factors stored in the dentin thereby influencing bone formation. To test this hypothesis, collagen membranes were either soaked in acid dentin lysates (ADL) from extracted porcine teeth or serum–free medium followed by lyophilization. Thereafter, these membranes covered standardized 5-mm-diameter critical-size defects in calvarial bone on rats. After four weeks of healing, micro-computed tomography and histological analyses using undecalcified thin ground sections were performed. Micro-computed tomography of the inner 4.5 mm calvaria defects revealed a median bone defect coverage of 91% (CI: 87–95) in the ADL group and 94% (CI: 65–100) in the control group, without significant differences between the groups (intergroup *p* > 0.05). Furthermore, bone volume (BV) was similar between ADL group (5.7 mm^3^, CI: 3.4–7.1) and control group (5.7 mm^3^, CI: 2.9–9.7). Histomorphometry of the defect area confirmed these findings with bone area values amounting to 2.1 mm^2^ (CI: 1.2–2.6) in the ADL group and 2.0 mm^2^ (CI: 1.1–3.0) in the control group. Together, these data suggest that acid dentin lysate lyophilized onto collagen membranes failed to modulate the robust bone formation when placed onto calvarial defects.

## 1. Introduction

Bone augmentation is a standard procedure to overcome the atrophy of the alveolar bone upon tooth extraction, with the overall aim to rebuild a bony support for the placement of dental implants [1,2]. Initially, augmentation procedures often involved the use of autologous bone obtained from the same patient, either alone or combined with bone substitutes to subsequently allow the placement of dental implants [2]. Autologous bone is a favorable graft because of its osteoconductive properties [3], osteogenic activity [4,5], and source of growth factors stored in the matrix [6]. However, the limited amount of local autologous bone and the morbidity upon harvesting [7] has stimulated the search for alternative sources of these bone substitutes including allografts and xenografts. Allografts [8] and xenografts [9] are common bone substitutes in clinical practice and they are widely used for bone augmentation. These substitutes, however, are rather passive in their capacity to stimulate the early stages of graft consolidation [3]. Therefore, other alternative sources of autografts have been recently proposed, namely dentin.

The autologous demineralized dentin matrix was intensively investigated by the group of Kim et al. [10,11,12], while Schwarz et al. were in favor of mineralized autogenous tooth roots [13,14,15,16,17]. Histological analysis revealed a resorption and substitution of autogenous tooth roots by a non-mineralized, well-vascularized tissue zone, which was gradually invaded by a network of woven bone in a canine model [18]. Considering that dentin, similar to bone [6], is a rich source of growth factors that are released by osteoclasts during resorption [19], it seems relevant to determine the impact of acid dentin lysates (ADL) on bone regeneration.

Based on a similar approach, we have recently identified that acid bone lysate is a rich source of TGF-β [6], and that lyophilization of acid bone lysates onto collagen membranes decreased bone regeneration in rat calvarial defects [20]. Likewise, we have recently identified TGF-β as a major growth factor in ADL by a combined proteomics and RNA sequencing approach (Nasirzade et al. under revision). More recently, our group, showed that ADL induces an inflammatory response when being prepared from uncleaned teeth [21]. This inflammatory response, nevertheless, was not triggered when teeth had been cleaned with toothpaste and a toothbrush [21]. Therefore, it appears that ADL is capable of causing a strong cellular response in vitro, however, the translation of these findings into a preclinical scenario remains largely unknown.

The rational of the study was based on the hypothesis that growth factors released from dentin by acid osteoclastic lysis could modulate bone regeneration and eventually enhance graft consolidation. The aim of the present study was, therefore, to examine the impact of ADL lyophilized onto collagen membranes on bone regeneration. We report here that the robust bone formation in rat calvaria defects was not disturbed by ADL lyophilized onto collagen membranes.

## 2. Material and Methods

### 2.1. Study Design

The current study was performed at Department of Biomedical Research in Medical University of Vienna in accordance with ARRIVE guidelines [22] and approved by the local ethical committee at the Medical University of Vienna (GZ BMWFW-66.009/0217-WF/V/3b/114/2012.20). A total of 20 adult (200–300 g) female Sprague–Dawley rats (Division for Biomedical Research, Himberg, Austria) were randomly assigned to receive collagen membranes (Bio-Gide^®^, Geistlich, Wolhusen, Switzerland) that had been soaked either in ADL (ADL group) or in serum-free medium (control group). Following freezing at −80 °C, lyophilization was performed (Alpha 1-2 LDplus, Martin Christ, Osterode am Harz, Germany).

### 2.2. Acid Dentin Lysate

Teeth were extracted from adult pigs within 6 h post-mortem (Fleischerei Leopold Hödl, Vienna, Austria). Extracted teeth were cleaned from periodontal ligaments and soft tissue attachment with a surgical blade (Swann-Morton, Sheffield, UK) and then enamel was removed by using a manual grinding and polishing device (Metaserv 2000, Cleveland, OH, USA). Subsequently, the pulp chamber was cleaned with a dental probe (Instrapac, Worksop, UK) and the teeth were crushed using a hammer. One gram of wet crushed dentin pooled from around five teeth was incubated with 10 mL of 0.1 N HCl (10% weight/volume) while being stirred overnight at room temperature. The resulting acid dentin lysate (ADL) was then centrifuged and the pH of the supernatant was neutralized. Following sterile filtration, ADL was kept frozen at −20 °C. The stock was thawed immediately before the experiment.

### 2.3. Surgical Procedures and Postoperative Treatment

The housing and husbandry of the animals were according to the local guidelines for animal care with free access to water and a standard diet [23]. Surgeries were performed as previously described [24]. In brief, all animals received ketamine (50 mg/kg i.p., AniMedica, Senden, Erlangen, Germany) and xylazine hydrochloride (10 mg/kg i.p., Bayer Austria, Vienna, Austria). One unilateral, bicortical, mid-parietal defect of 5-mm-diameter was generated on the parietal bone between the frontal suture and the sagittal suture of the calvaria by a trephine burr (Dentium Inc., Seoul, Korea). After randomization, the collagen membranes (either soaked in ADL or serum-free medium) were trimmed and placed onto the defects. The membrane overlapped the walls of the defect by at least 1 mm. Thereafter, the membranes were stabilized and the flap was sutured in layers with resorbable sutures (Vicryl 5-0; Ethicon GmbH, Norderstedt, Germany). Buprenorphine 0.06 mg/kg, (Temgesic^®^, Temgesic, Reckitt, and Colman Pharm., Hull, UK) and piritramide were administered as analgesics. After a healing period of four weeks, animals were euthanized using intracardial overdose of sodium pentobarbital (300 mg/kg). Then, all specimens were processed for micro-computed tomographic (μCT) and histological analysis.

### 2.4. Immunoassay of TGF-β

Independent preparations of ADL were assessed to measure the concentration of TGF-β1. Immunoassay of TGF-β1 was performed according to the manufacture’s instruction using a quantitative kit (R&D System, Minneapolis, MN, USA).

### 2.5. Micro CT Analysis

The heads were fixed in phosphate-buffered formalin (Roti-Histofix 4%, Carl Roth, Karlsruhe, Germany). Micro-CT was performed at 90 kV/200 μA with an isotropic resolution of 20.7 μm and an integration time of 500 ms (μCT 50 Scanco Medical AG, Bruttisellen, Switzerland). Three-dimensional reconstruction of the data was performed using the visualization software Amira 6.2 (Thermo Fisher Scientific, Waltham, MA, USA). The image analysis was performed by uploading the images stacks and then rotated using Fiji [25] to obtain the drill direction in the Z axis with the defect near the center of the image. The region of interest (ROI), corresponding to the defect diameter, was positioned manually and automatically segmented from the µCT images which included the entire thickness of the calvarial bone with an individually developed ruleset. The inner 4.5 mm of the defect area that had the high-density of mineralized tissues was the basis for the analysis of the bone defect coverage and new bone volume.

### 2.6. Histological and Histomorphometric Analysis

All samples underwent dehydration using increasing grades of alcohol and embeded in light-curing resin (Technovit 7200 VLC + BPO, Kulzer and Co., Wehrheim, Germany). Samples were further handled using a cutting and grinding equipment (Exakt Apparatebau, Norderstedt, Germany). Thin- ground sections of around 100 µm were prepared in a plane with the sagittal suture and through the center of the defect, before being stained with Levai-Laczko dye, a variant of the Giemsa dye that allows to distinguish reliably between old bone and new bone [26].

The slices were scanned using an Olympus BX61VS digital virtual microscopy system (DotSlide 2.4, Olympus, Tokyo, Japan) with a 20× objective resulting in a resolution of 0.32 µm per pixel and then were segmented manually using Photoshop CS 4 (Adobe Systems Inc., San Jose, CA, USA). The segmented images were then measured in Fiji using the Bone J plugin based on the color-coded thresholding that was obtained from the correspondent histograms [27]. The histomorphometric analysis was performed at three regions of interest (ROIs) representing the central compartment within the defect margins, the adjacent ectocranial compartments, and the outer compartment on the surface of the host’s cortical bone.

### 2.7. Statistics

Statistical analysis was based on the data observed with the µCT and histomorphometric analysis. For µCT, median values and confidence intervals (CI) of the primary outcome (% of bone defect coverage) and the bone volume between control and ADL group were compared with Mann–Whitney U test. For histomorphometry, bone area between control and ADL group were also compared with Mann–Whitney U test. Analyses were performed using Prism v8 (GraphPad, La Jolla, CA, USA). Owing to the pilot nature of the study, the sample size was chosen based on experience from previous studies [24] to balance the ability to measure significant differences while reducing the number of animals used. Significance was set at *p* < 0.05.

## 3. Results

### 3.1. TGF-β Immunoassay

Results from our proteomic approach revealed TGF-β as one of the main growth factors presented in ADL (Nasirzade et al. under revision). Data from the immunoassay showed TGF-β1 with a concentration range of approximately 0.8 to 2.8 ng/mL, in nine independent preparations of ADL.

### 3.2. µCT Analysis

**Figure 1:**
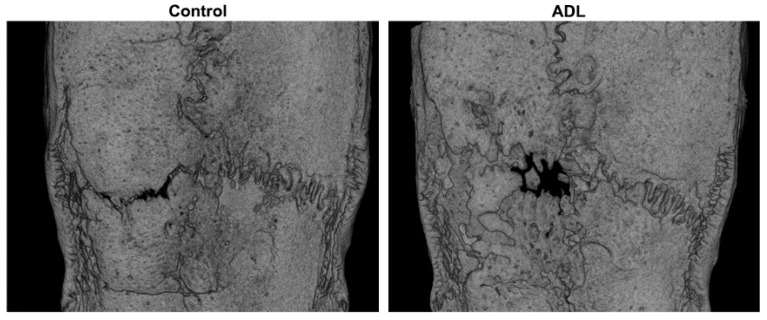
Micro-CT overview of the defect anatomy shows almost complete coverage after four weeks of healing. Rat calvaria defects with opening of the sutures were covered with a collagen membrane either soaked in serum-free medium (**Control**), or in acid dentin lysates (**ADL**). The images represent the specimens with a median defect coverage.

**Figure 2:**
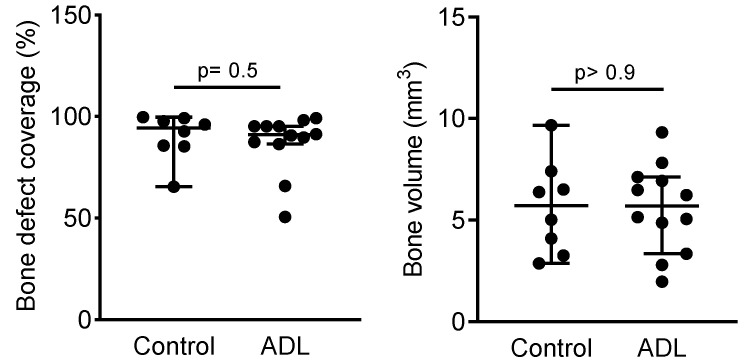
Acid dentin lysate (ADL) failed to affect bone regeneration in the calvaria defect. Quantitative analysis of bone defect coverage (%) and bone volume (mm^3^) in the inner 4.5 mm of the defect. The two groups were compared with Mann–Whitney U test and *p* values are indicated.

Figure 1 represents the aerial view of the median defect coverage, clearly demonstrating the solid bone bridging in both control and ADL group. Quantitative analysis of the inner 4.5 mm of the defect demonstrated a bone defect coverage in ADL group of 91% (CI: 87–95) and of 94% (CI: 65–100) in control group (*p* > 0.05) (Figure 2). Likewise, the bone volume in the defect was 5.7 mm^3^ (CI: 3.4–7.1) in control group and 5.7 mm^3^ (CI: 2.9–9.7) in ADL group (*p* > 0.9) (Figure 2). Other parameters of the µCT analysis such as trabecular thickness and trabecular separation also revealed no difference between the two groups (data not shown).

### 3.3. Histomorphometric Analysis

**Figure 3:**
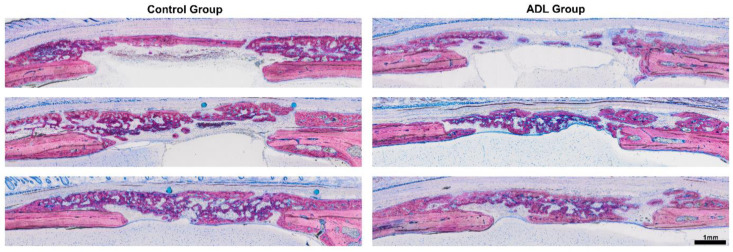
Histological overview of the defect anatomy after four weeks of healing. Rat calvaria defects were treated with native collagen membranes soaked either in serum-free medium, or in acid dentin lysate (ADL). Histological pictures representing the samples with minimum (upper images), median (middle images) and maximum (lower images) bone area based on quantitative analysis. The local host calvaria bone demarcates the defect borders and appears in light purple. The newly formed bone is stained in dark purple. Undecalcified thin ground sections stained with Levai-Laczko.

**Figure 4:**
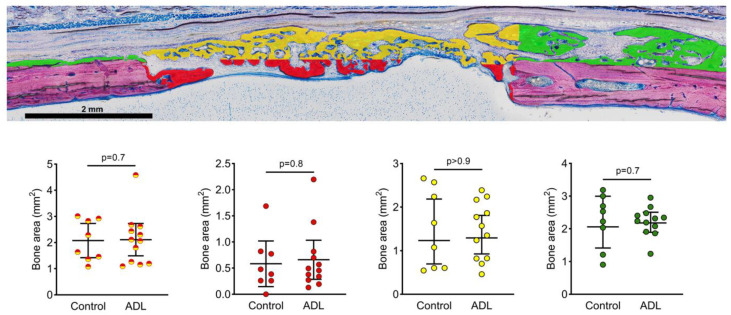
Histomorphometric analysis of the bone area in the defect after four weeks of healing. Histomorphometry was performed at three regions; (red) the central compartment within defect margins, (yellow) the adjacent ectocranial compartments, and (green) the outer compartment on the surface of the host cortical bone. Red-yellow represents the sum of both groups. The groups were compared with Mann–Whitney U test and *p* values are indicated.

A histomorphometric analysis confirmed the lack of clear differences between the groups (Figure 3). In the central compartment and the adjacent ectocranial compartment within the defect margins, the bone area in the control group was 2.1 mm^2^ (CI: 1.2–2.6) and in ADL group 2.0 mm^2^ (CI: 1.1–3.0) (*p* > 0.05). Less new bone was observed in the central defect site when compared to the ectocranial compartment (Figure 4). The outer compartment on the surface of the host cortical bone showed considerable bone formation, again not being affected by ADL (Figure 4).

### 3.4. Histological Analysis

**Figure 5:**
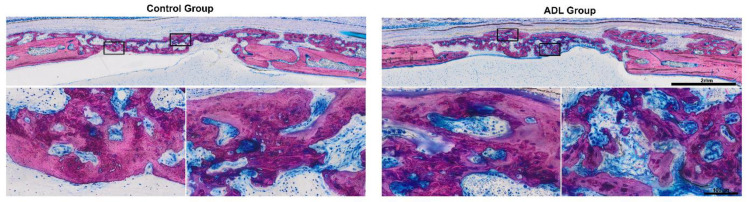
Detailed overview on the new bone in the control group and in the acid dentin lysate (ADL) group. Note the characteristic features of immature woven bone indicated by the intense purple stain and the large osteocyte lacunae. The dense part of the membrane is visible in the upper part of control group also showing that new bone grows on the collagen membrane.

At the level of the descriptive histology, ADL had no impact on the overall picture. In both groups, bone formation occurred actively within the collagen membrane (Figure 5) [24]. The fibers of the original collagen membrane (light pink) were either surrounded by the new bone or soft tissue. The calvarial defect in the control group was mainly filled by woven bone (dark purple). This woven bone formed trabecular ridges with random orientation and was enclosed either by thin layers of parallel-fibered bone (light purple) or thin layers of osteoid.

## 4. Discussion

This research was inspired by the notion that growth factors released from dentin upon osteoclastic resorption, simulated by an acid lysis procedure [6], affect the process of bone regeneration. We took advantage of our well-established model where collagen membranes served as carriers for the bioactive molecules and as scaffolds to lay down new bone in a rat calvaria defect [20,24]. Based on our previous observations with acid bone lysates [20], we were expecting a reduction of newly formed bone by ADL. However, using two different methods of analysis, µCT and the histomorphometry, the results revealed that ADL had no significant impact on the osteoconductive properties of the collagen membrane.

If we relate these findings to those of others, our data are in line with the large variation within each group as recently reported for bone conditioned medium and acid bone lysate [20,24]. Our data also support the observations with bone conditioned medium where no considerable changes on bone regeneration were reported. However, acid bone lysate, which is rather comparable to ADL, significantly reduced bone regeneration under similar conditions [20]. Moreover, the histological appearance of the newly bone formed, particularly the collagen fibers being entombed by the new bone, was comparable to previous studies [20,24]. Further support comes from the previous observations indicating that the collagen membrane in particular somewhat promotes the bone formation outside the borders of the defect margins [20,24].

The clinical relevance of the present study leaves room for speculations. Possibly independent of the original research question, we have confirmed the favorable osteoconductive properties of the collagen membrane along with the robustness of the pre-clinical model. More importantly, however, is the lack of a significant impact of ADL on bone regeneration. This does not necessarily indicate that growth factors released upon resorption of autogenous tooth roots have no impact on bone regeneration [18]. In fact, one might speculate that the favorable properties of the collagen matrix may overshadow the possible beneficial effects of ADL, or, in contrast, that the ADL is not strong enough to reduce the robust bone regeneration observed in this study. Recent histological observations even showed that it is the demineralized dentine that shows favorable bone formation activity compared to the whole-tooth in augmentation sites [26]. Thus, it might be that the relevant growth factors supporting bone regeneration remain in the demineralized dentine and are not necessarily found in the acid dentine lysate. Another interesting clinical aspect for future studies could be the measurements of biomarkers, such as TGF-β1, VEGF, and soluble urokinase-type plasminogen activator receptor. These biomarkers can bind to collagen membranes possibly influencing the outcome of graft consolidation [28,29].

Clearly there are limitations that should be acknowledged. First, the study has to be considered as a pilot study since we have not included earlier and more time points. In this sense, it cannot be ruled out that effects of ADL are no longer detectable after a one-month observation period. Similarly, whether there is a dose-response relationship remains unknown. Second, a large variation within both groups reduces the likelihood to observe significant changes. Future studies might use a more homogenous carrier to lower the variance; however, this material needs to be established. Moreover, the bone originating from the sutures might have overshadowed possible effects of ADL, which was not the case in our previous report where the sutures remained untouched [20]. Third, the acidic treatment does not necessarily recall the complex process of osteoclast resorption that combines an acidic lysis with a proteolytic cleavage of the extracellular collagen-rich matrix [30]. Thus, ADL preparation can only partially simulate the paracrine environment of dentin that undergoes resorption. Fifth, our model does not represent a clinical situation where autologous teeth are used and there is even the chance that the ADL produced by porcine dentin is unsuitable for the present rat model, having a xenogenic situation of transplantation. Thus, care should be taken when interpreting the present finding and any overintepretation should be avoided.

In summary, we report here that the favorable osteoconductive properties of a widely used native collagen membrane are not significantly affected by their soaking and lyophilization in acid dentin lysate on a critical-size defect model.

## Data Availability

The data sets used and/or analyzed during the current study are available from the corresponding author or reasonable request.
